# Dry-fog decontamination of microbiological safety cabinets after activities with SARS-CoV-2: cycle development and process validation for dry fogging with peroxyacetic acid

**DOI:** 10.3205/dgkh000397

**Published:** 2021-08-31

**Authors:** Jens Peter Teifke, Hendrik Scheinemann, Jan Schinköthe, Michael Eschbaumer, Alina Melüh, Mathias Streitz, Holger Freese, Sven Reiche

**Affiliations:** 1 Department of Experimental Animal Facilities and Biorisk Management, Friedrich-Loeffler-Institut, Federal Research Institute for Animal Health, Greifswald-Insel Riems, Germany; 2 Institute of Diagnostic Virology, Friedrich-Loeffler-Institut, Federal Research Institute for Animal Health, Greifswald-Insel Riems, Germany

**Keywords:** disinfection, aerosolization, biological safety cabinet, biological agent, occupational safety

## Abstract

**Background:** Technical protection measures for laboratory activities involving biological agents include biological safety cabinets (BSC) that may be contaminated. In the case of diagnostic activities with SARS-CoV-2, this may also affect BSC that are operated at protection level 2; therefore, decontamination of all contaminated surfaces of the BSC may be required. In addition to fumigation with hydrogen peroxide (H_2_O_2_), dry fogging of H_2_O_2_-stabilized peroxyacetic acid (PAA) represents another alternative to fumigation with formalin. However, to prove their efficacy, these alternatives need to be validated for each model of BSC.

**Methods:** The validation study was performed on 4 different BSCs of Class II A2 using the “Mini Dry Fog” system.

**Results:** An aerosol concentration of 0.03% PAA and 0.15% H_2_O_2_ during a 30 min exposure was sufficient to inactivate SARS-CoV-2. Effective concentrations of 1.0% PAA and 5% H_2_O_2_ were required to decontaminate the custom-prepared biological indicators loaded with spores of *G. stearothermophilus* and deployed at 9 different positions in the BSC. Commercial spore carriers were easier to inactivate by a factor of 4, which corresponded to a reduction of 10^6^ in all localizations.

**Conclusions:** Dry fogging with PAA is an inexpensive, robust, and highly effective decontamination method for BSCs for enveloped viruses such as SARS-CoV-2. The good material compatibility, lack of a requirement for neutralization, low pH – which increases the range of efficacy compared to H_2_O_2_ fumigation – the significantly shorter processing time, and the lower costs argue in favor of this method.

## Zusammenfassung

**Hintergrund: **Zu technischen Schutzmaßnahmen bei Labortätigkeiten mit Biostoffen gehören auch mikrobiologische Sicherheitswerkbänke (MSW), die durch Biostoffe kontaminiert sein können. Dies kann bei diagnostischer Tätigkeit mit SARS-CoV-2 auch solche MSW betreffen, die in der Schutzstufe 2 betrieben werden. Für die technische Freigabe kann eine Dekontamination aller mikrobiologisch belasteten Oberflächen der MSW erforderlich sein. Neben der Begasung mit Wasserstoffperoxid (H_2_O_2_) stellt die Trockenvernebelung von H_2_O_2_-stabilisierter Peroxyessigsäure (PES) eine weitere Alternative zur Begasung mit Formalin dar. Um die Wirksamkeit zu belegen, müssen allerdings diese Alternativen an den jeweiligen MSW-Modellen validiert werden.

**Methode:** Die Validierungsstudie wurde an 4 verschiedenen MSW Klasse II unter Verwendung des „Mini Dry Fog“-Systems durchgeführt.

**Ergebnisse:** Für die Inaktivierung von SARS-CoV-2 reichte eine Aerosolkonzentration von 0,03% PES und 0,15% H_2_O_2_ über 30 min Einwirkzeit aus. Zur Dekontamination der mit Sporen von *Geobacillus* *stearothermophilus* selber beschickten und an 9 unterschiedlichen Positionen in der MSW ausgebrachten Keimträger waren Wirkkonzentrationen von 1,0% PES und 5% H_2_O_2_ erforderlich. Kommerziell erhältliche Sporenkeimträger waren um den Faktor 4 empfindlicher, was in allen Positionen einer Reduktion um 10^6^ entsprach.

**Schlussfolgerung:** Trockenvernebelung von PES ist ein preisgünstiges, robustes und für behüllte Viren wie SARS-CoV-2 hoch wirksames Dekontaminationsverfahren für MSW. Die hohe Materialverträglichkeit, die nicht erforderliche Neutralisation, der niedrige pH, durch den im Vergleich zur H_2_O_2_-Begasung das Wirkungsspektrum erweitert wird, die deutlich kürzere Prozessdauer und geringere Investitionskosten sprechen für dieses Verfahren.

## Introduction

### Biological safety cabinets

Biological safety cabinets (BSC; microbiological safety workbenches, MSW) are among the technical protective measures in microbiological laboratories that protect users, the working area and the environment, as well as the materials being handled from the harmful effects of biological agents [1]. Based on DIN EN 12469:2000-09, Class II A2 BSCs, which are typically also used in microbiological diagnostic laboratories, serve to protect personnel and products and prevent carry-over [2]. During activities involving biological agents, a contamination of the interior of a BSC cannot be ruled out. This can affect not only the actual workspace, but also the HEPA filters exposed to biological agents. In the event of improper operating conditions, e.g., leakage or compromised seal of the filters, the air-handling components required to generate the laminar air flow, such as fans and flow ducts, can also be contaminated [3]. To ensure a safe working environment, BSCs must be inspected not only after installation, but also after each significant, safety-relevant modification or maintenance measure, such as relocation or filter replacement. This applies to all BSCs, at regular (usually annual) intervals. For this purpose, it is necessary that the operator issue a written work authorisation based on a validated decontamination procedure [1]. 

### Disinfection and decontamination

Prior to any work on a BSC, such as periodic inspection, maintenance or repair, but also when disposing of components or of the entire BSC, decontamination with a biocidal product suitable for this purpose and approved by the competent authority is mandatory at protection level 3 (BSL3) and higher. The efficacy of the procedure must be validated [1], [3]. The question of whether, when and how such decontamination is necessary already arises during the authorisation procedure pursuant to Section 15 (3) of the Biological Agents Ordinance. However, in the context of the ongoing SARS-CoV-2 pandemic, the performance of non-specific diagnostic activities [4] may make it necessary to perform decontamination procedures even at protection level 2 (BSL2). Their effectiveness must be proven and documented before release of the BSC to the service personnel [1]. Finally, it must be determined whether disinfection of all surfaces exposed to biological agents within the BSC is necessary and if and how filters are to be replaced [3]. Based on the requirements of the Technical Rules for Biological Agents (TRBA) 100, the following approved procedures can be used when changing the filter of a BSC: a) the interior of the BSC and the HEPA filter are fumigated in situ with formaldehyde to reduce the biological load (room disinfection procedure according to TRGS 522), or b) the interior and HEPA filter are decontaminated in situ with a validated fumigation procedure using hydrogen peroxide [5]. After formaldehyde fumigation, the HEPA filters must be sterilised using moist heat before final disposal. In contrast, after treatment with vaporised H_2_O_2_, the decontaminated HEPA filters may be disposed of as non-infectious waste [5]. For H_2_O_2_ fumigation of BSCs, precisely specified process parameters, suitable gas generators and BSC specifications (volume, HEPA filter material) are listed in the “List of disinfectants and disinfection procedures tested and approved by the Robert Koch Institute in accordance with Section 18 of the Infection Protection Act”, which can also be used as a guideline for laboratory operations [6]. Procedures deviating from this standard always require process validation [3].

Formaldehyde fumigation can be used to meet disinfection requirements after appropriate validation, but room disinfection by formaldehyde evaporation may only be carried out by persons who have a certificate of competence and a permit from the competent authority (in accordance with the Ordinance on Hazardous Substances and TRGS 522). In addition to the need to neutralise it after the process, formaldehyde has other negative properties, which will increasingly limit its use for disinfection purposes in the future [1]. When selecting decontamination processes and disinfectants, the protection of people and the environment must be taken into account, and the most gentle, yet effective, biocidal product available must be used in each case. In this context, the provision and use of disinfectants as biocidal products is fundamentally governed by Regulation (EU) No. 528/2012 (Biocide Regulation).

In addition to, and increasingly as a substitute for, formaldehyde fumigation and as an alternative to H_2_O_2_ fumigation, the “dry fog” method is used for decontamination of rooms, including BSCs [7], [8], [9]. This method is based on the aerosolisation of disinfectants through the application of compressed air. The ultra-fine droplet size of 7.5 µm on average generated by the “Venturi effect” means that the dry fog remains in suspension as an aerosol for a very long time and condenses only minimally. Due to its excellent, broad microbicidal properties, which are also present at low temperatures and extremely low concentrations, peroxyacetic acid (PAA, peracetic acid, C_2_H_4_O_3_) is particularly suitable for this process [1]. Dry fogging largely obviates the corrosive effects of PAA. In cleanroom technology, regarding disinfection of operating theatres, highly complex technical facilities and high-security laboratories, room volumes between 1 m^3^ and 1,000 m^3^ can be disinfected, depending on the equipment used (literature in [10]). Under optimal conditions of relative humidity (rH) and temperature (T), such fogging can achieve effective decontamination by reducing the infectivity of viruses and bacteria, including spores, by six powers of ten (10^6^) [7], [9]. The main advantages of this robust method, which is largely based on the oxidative properties of PAA chemically stabilised by hydrogen peroxide, are the short exposure time, the high material compatibility at low effective concentrations for a broad spectrum of biological agents, and the relatively inexpensive and easy-to-handle equipment required. Validation data for the decontamination of BSCs by means of H2O2 fumigation have been previously published [11], [12]. The dry-fog decontamination of laboratory rooms with PAA has been described, but there is only limited experience concerning BSCs [13].

For this reason, we describe the cycle development and process validation for different BSCs, using a previously validated surrogate for SARS-CoV-2.

## Materials and methods

Four different **BSCs** of Class II were available for the validation study and process development: Berner FlowSafe B-[MaxPro]^2^-130, Holten LaminAir Safe 2010 Model 1.5, Tecniplast BS48 and Thermo Electron HERAsafe KS18. 

**Figure 1:**
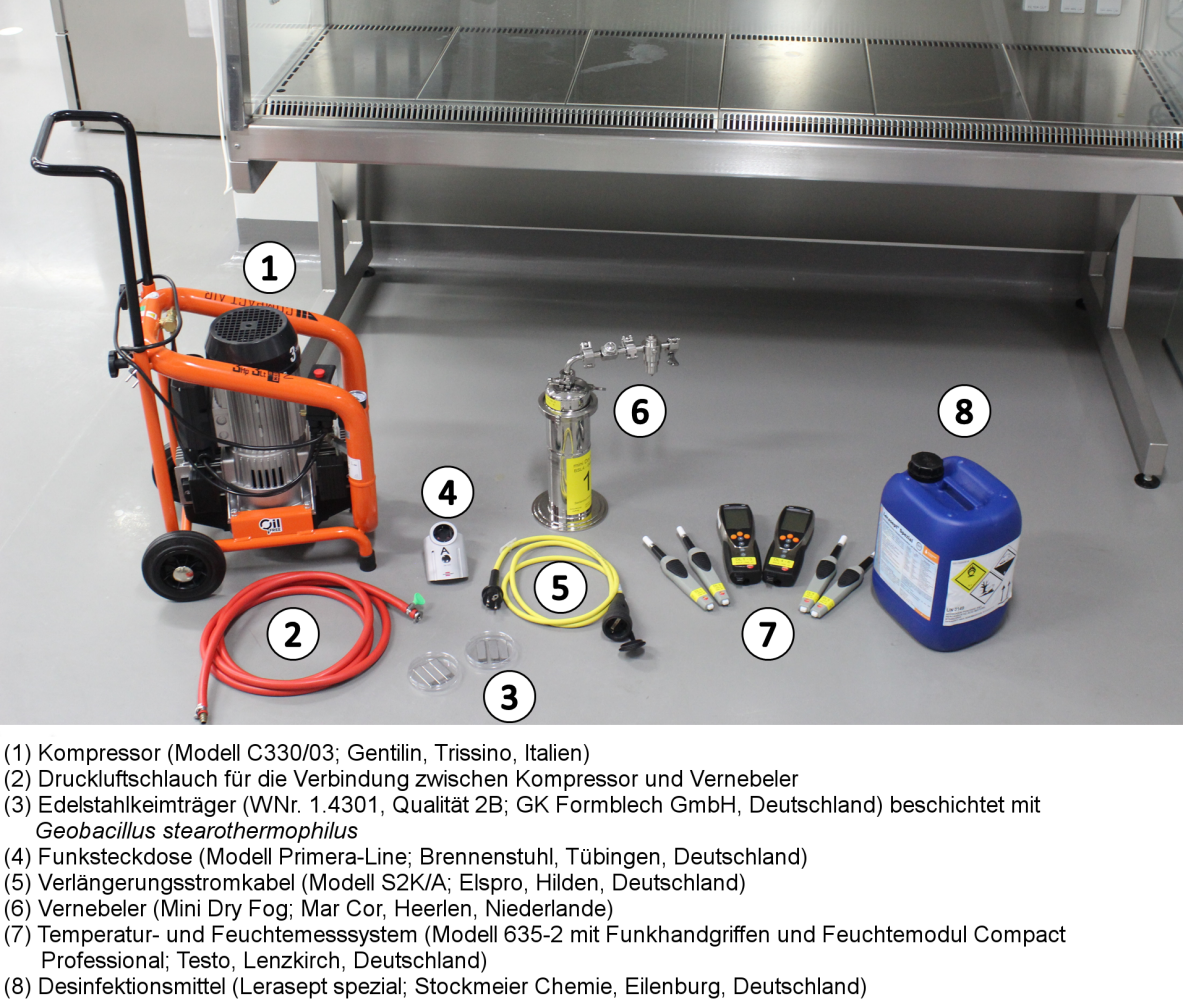
Equipment required for PAA dry fogging of a BSC

The **dry fogging system** “Mini Dry Fog” used for decontamination and the additionally required equipment are shown in Figure 1 (Fig. 1). The fogger was always placed on the right side within the workspace of the BSC and its nozzle was pointed towards the left side at an angle of about 20° above horizontal.

By diluting the listed [14] **biocidal product** “Lerasept Spezial” (an equilibrium mixture of PAA, acetic acid and H_2_O_2_) in fully demineralised water, a working solution at a concentration of 1.3% PAA and 6.8% H_2_O_2_ was obtained.

By means of an oil-free **air compressor** located outside the BSC, at least 0.3 MPa overpressure at an air flow rate of at least 70 l/min was generated for aerosolisation of the biocidal product. The air hose was inserted through an opening in the side wall of the BSC where possible, otherwise through the working opening.

Inactivation kinetics for SARS-CoV-2 were determined in **previous studies** as described [9]. As a surrogate, spores of *Geobacillus stearothermophilus* were selected in preference to *Bacillus subtilis*, due to the high tenacity and more practical evaluation by selective cultivation at 60°C [9]. Commercially available and custom-coated spore carriers were compared. It was shown that a PAA concentration of 0.0313% was sufficient for successful disinfection (≥10^4^-fold reduction) of SARS-CoV-2. In order to achieve a comparable disinfection effect of the commercially available spore carriers, on the other hand, an approximately eightfold higher PAA concentration of 0.25% was required. At the same time, the commercial germ carriers showed a significantly higher sensitivity to PAA aerosol compared to the germ carriers coated with spores of *G. stearothermophilus*, which required a PAA concentration of 1.0% [9], approximately 32 times higher than SARS-CoV-2.

For this reason, the custom-prepared germ carriers with *G. stearothermophilus* spores were selected as a surrogate for SARS-CoV-2 for the validations described here, providing a safety margin. Hot-air sterilised 16×60 mm stainless steel strips were coated with 10^6^/50 µl spores of *G. stearothermophilus* in PBS as a defined smear (10 µl/cm^2^) according to EN 10088-2 and dried. In addition to the exposed germ carriers, another germ carrier was not exposed to the disinfectant aerosol and used as a desiccation (negative) control. 

After completion of a fogging cycle, the treated spores were rinsed from the germ carriers with liquid nutrient medium, and incubated in a serial ten-fold dilution series for 7 days at 60°C. In this manner, the remaining amount of reproducible spores was determined. The reduction in infectivity was calculated in comparison with the non-treated desiccation control.

**Figure 2:**
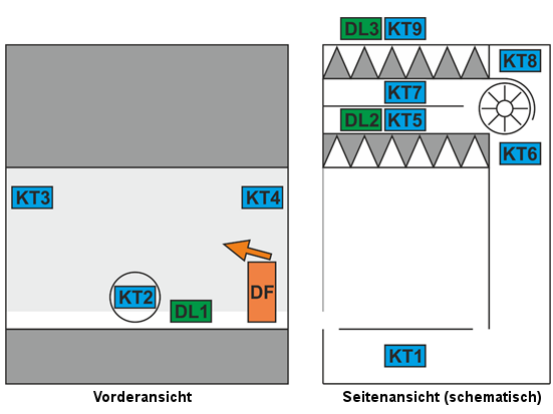
Position of the “Mini Dry Fog” device (DF, orange; arrow shows spray direction of nozzle), data loggers (DL 1-3, green) and germ carriers (GC, blue) in the BSC; Biological indicator localisations are: GC1: lower exhaust plenum; GC2: in closed Petri dish in workspace; GC3: workspace, left side; GC4: workspace, right side; GC5: upstream side recirculation filter; GC6: exhaust plenum, in airflow; GC7: upstream of exhaust filter; GC8: exhaust plenum, “dead angle”; GC9: downstream of exhaust filter

For the three independent runs of the validation, 9 germ carriers and 3 measuring and recording devices for temperature and humidity (data loggers) were placed in the respective BSC. Portable **measuring devices with data loggers** were used for wireless monitoring during decontamination. In order to be able to place the germ carriers and data loggers, the exhaust plenum of the BSC had to be opened and the HEPA filters also had to be briefly removed. For the validation runs, the filters were reinserted and the plenum was sealed. Following the validation, a functional test of the BSC was carried out in each case, including a retention efficacy and leak test of the HEPA filters. The positioning of the “Mini Dry Fog” device, the germ carriers and data loggers can be seen in the schematic in Figure 2 (Fig. 2). The position “KT9” on the downstream side of the exhaust air filter is considered to be a critical point for all operating conditions, because experience has shown that it is the most difficult for disinfectants to reach [3].

The following process cycle was established and applied:

1. **Conditioning phase I**: Aerosolisation of approx. 20 ml/min PAA in “standby” or “night” operation mode; exhaust vent (KT9) covered with plastic sheeting and sealed with tape on three sides; front sash in lowest position (depending on design, not all BSCs could be operated with the sash completely closed), for 20 min; increase in relative humidity (rH) in the work area to 99.9% (saturation).
2. **Conditioning phase II**: Switch off the BSC, completely seal the exhaust vent and, if necessary, seal the front sash with industrial adhesive tape; after a further 2 min of fogging, switch off the compressor.
3. **Decontamination phase**: Exposure to PAA in the closed BSC interior for 30 min.
4. **Ventilation phase**: Uncover the exhaust vent and run the BSC for 60 min at full air flow and standard sash position to remove the biocidal product. Subsequently, germ carriers and radio sensors were removed for evaluation.

The essential parameters of the process cycle are shown in Table 1 (Tab. 1).

## Results

**Figure 3:**
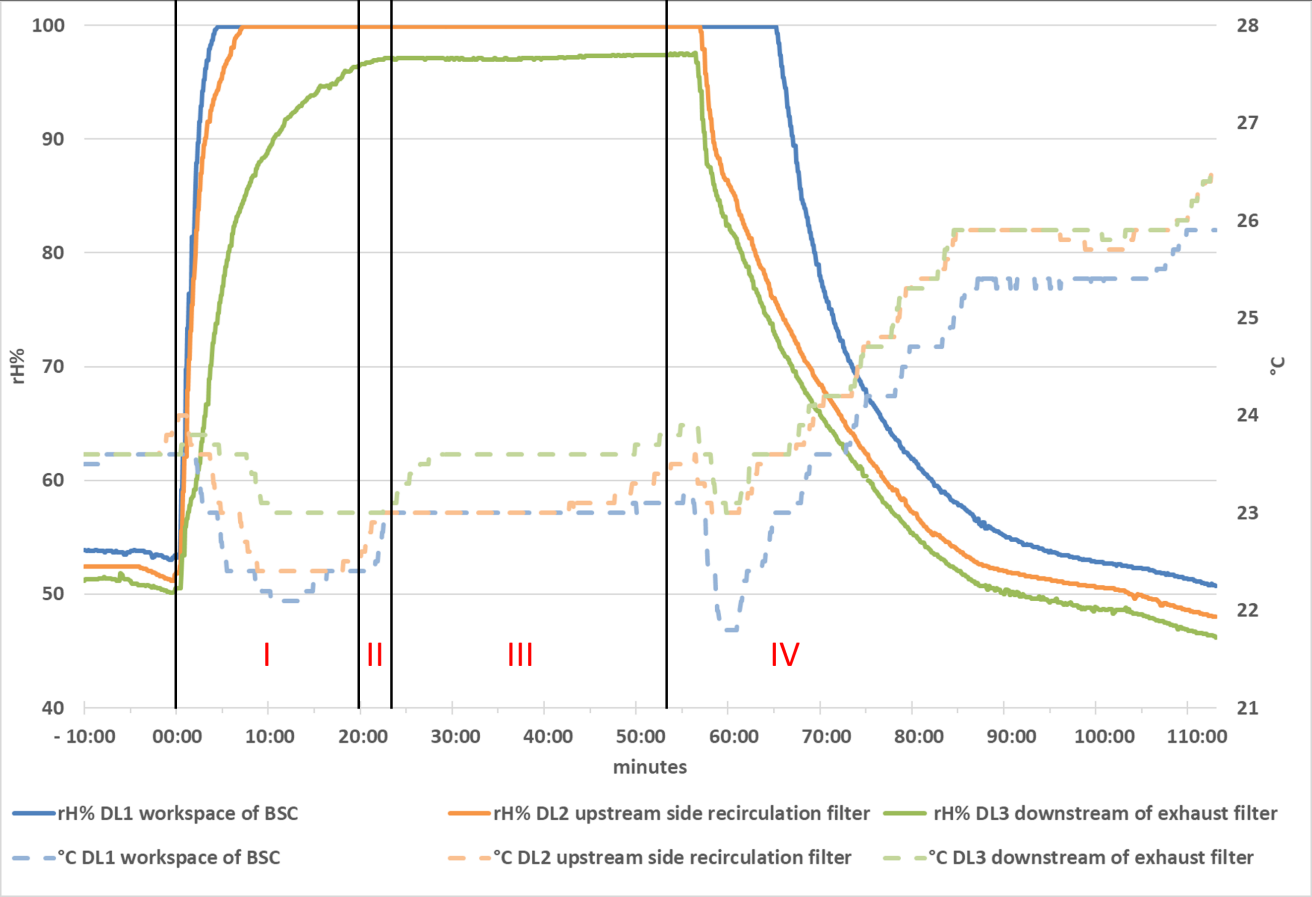
Illustration of the process phases (I: conditioning phase 1; II: conditioning phase 2; III: decontamination phase; IV: ventilation phase) and the measured relative humidity (rH in %) and temperature (T in °C) at three positions of data loggers (DL 1-3) using the example of fogging in the Holten LaminAir Safe 2010 Model 1.5.

Previous tests with SARS-CoV-2-coated germ carriers applied in the working area of the BSC showed that an aerosolised concentration of 0.03% PAA and 0.15% H_2_O_2_ and an exposure time of 30 min are sufficient to reduce infectivity by 4 orders of magnitude [9]. However, for decontamination of the germ carriers loaded with *G. stearothermophilus* as a surrogate “with a safety margin”, approximately eight times higher effective concentrations of about 0.25% PAA and 1.25% H_2_O_2_ were required for commercially available germ carriers, or 1.0% PAA and 5.0% H_2_O_2_ for custom-produced germ carriers. In principle, the acceptance threshold for validation was defined as a reduction of the bacterial count on the custom-loaded germ carriers by at least a factor of 10^4^ (i.e., by 99.99%). Based on the pre-tests, this corresponds to at least a 10^6^ reduction of commercially available spore carriers. The values for rH and T measured in detail and for the different BSC types and locations as well as the inactivation data obtained are summarised in Figure 3 (Fig. 3) and Table 2 (Tab. 2). Importantly, for all four BSC types, the filters passed all tests (retention and seal) after the validation runs.

## Discussion

Decontamination processes are intended to make surfaces and objects safe to handle. From a microbiological point of view, it is therefore a matter of returning biological substances to the basic contamination level that carries no health risks [1]. Depending on the initial contamination of a BSC, the effort required for this can vary. While in many cases simple wipe-down disinfection is sufficient for decontaminating work equipment in the BSC or the work surface itself, fumigation methods are mainly used for rooms, air-handling systems, and HEPA filters [3]. Hydrogen peroxide fumigation is already replacing the previously common room disinfection by formaldehyde [1]. 

The lower cost and time expenditure as well as ease of execution make dry fogging with PAA an extremely material-compatible decontamination procedure [10]. Unlike fumigation with vaporised formaldehyde, it does not require neutralisation. Due to its low pH, it has a higher efficacy than H_2_O_2_ alone for certain highly tenacious biomaterials, such as foot-and-mouth disease virus. Nevertheless, proper cycle development and process validation must be carried out before using this method. Critical parameters to be monitored in the process include relative humidity, air temperature, the nebulised amount of biocidal product in the corresponding room volume, and the local air currents [3]. The biological efficacy is a function of exposure time and concentration of the active substance. Other critical control values include the uniform distribution of the aerosol. This can be achieved by switching off and hermetically sealing the BSC interior including the HEPA filter. It must be recorded in real time by determining the rH and T kinetics at different locations in the BSC as part of the validation.

To control the flow of exhaust air containing PAA during conditioning phase I and to obtain a hermetic seal of the BSC during the decontamination phase, the exhaust vent was covered with plastic sheeting similar to an air vessel. This was first sealed on three sides, and then on all sides using industrial adhesive tape.

For the proof of efficacy of the inactivation method, suitable germ carriers must be used. Suitable surrogates are those biomaterials whose physical-biological properties (e.g., enveloped or non-enveloped viruses, vegetative or permanent forms of bacteria) most closely match those whose inactivation is to be demonstrated. For the efficacy testing of fumigation procedures, especially those using H_2_O_2_, *G. stearothermophilus* is used as test organism [6]. In contrast to wet chemical surface disinfection, dry inactivation methods do not require the addition of so-called disinhibitors in the microbiological analysis of the germ carriers due to the very low residual active substance concentrations on the treated surfaces. Also, in contrast to wet chemical disinfection processes, it is not possible or necessary to remove these disinfectant residues from the surfaces.

Only recently, 10^6^ spores of *G. stearothermophilus* on filter paper carrier material was recommended as the standard [3]. In particular, the comparable properties of the carrier material should allow easy transferability to HEPA filters. However, there are reasons that speak against such a choice [15], [16]. Filter paper is not ideal because it is very likely to absorb the disinfectant, concentrate it and release it over time, leading to false negatives, i.e., spurious inhibition of microbial growth. Also, cellulose is known to chemically react with gaseous H_2_O_2_, catalysing its degradation and thus lowering the room concentration [17], [18].

Absorptive effects are not seen with non-porous carrier materials, and in the current literature these materials are predominantly used. To ensure the robustness of the decontamination, we used stainless steel germ carriers for these validations, which, for the reasons mentioned above, are considerably more difficult to inactivate than paper carriers or other porous and absorbent filter materials.

In addition to placement within the workspace and especially at other locations of the BSC that are difficult to access and most difficult for the air flow to reach, germ carriers should at least be positioned on the downstream side of the exhaust air filter. Experience has shown that this position is the critical location of the BSC because it is the most difficult to reach [3]. This was also shown in the validations carried out here, with the lowest reduction of *G. stearothermophilus* infectivity (between 10^4.8^ and 10^6.4^) observed at this location.

Taking into account the safety margin for the surrogate and the already validated data for SARS-CoV-2 vs. *G. stearothermophilus* [9], a complete inactivation of SARS-CoV-2 can be assumed based on these results. The germ carriers placed in the workspace directly under the recirculating air filter were all inactivated on the order of 10^4.5^ to 10^7.1^, demonstrating the high, reproducible efficacy. 

## Conclusions

In summary, the process and cycle conditions presented for the decontamination of biological safety cabinets allow a reduction of infectivity of four to six orders of magnitude for viruses, vegetative bacteria and spores, especially of *G. stearothermophilus* as biological indicator.

## Notes

### Competing interests

The authors declare that they have no competing interests.

### Acknowledgements

The authors would like to thank in particular Dr. Bärbel Niederwöhrmeier for her critical review and helpful contributions to the discussion.

### Funding

This work was supported by BMVg, Zuwendungsbescheid E/E590/FZ005/FF005, in addition to basic funding from BMEL. The authors assure that they will provide data on this upon justified request.

### Ethics

This paper does not include studies on humans or animals.

### Dual Use Research of Concern (DURC)

The DURC implications of the described methodology were discussed in the Biorisk Committee of the FLI on 05 February 2018. No concerns were expressed.

### Authorship 

JPT, SR: conception and design of the paper

HF, AM, ME: conduct of the validation studies

HS, JS, ME, AM, MS, HF: data collection, analysis and interpretation

JPT: draft manuscript

SR, HS, JS, ME, MS: critical revision of the article

JPT, HS, JS, ME, AM, MS, HF, SR: final approval of the version intended for publication

## Figures and Tables

**Table 1:**
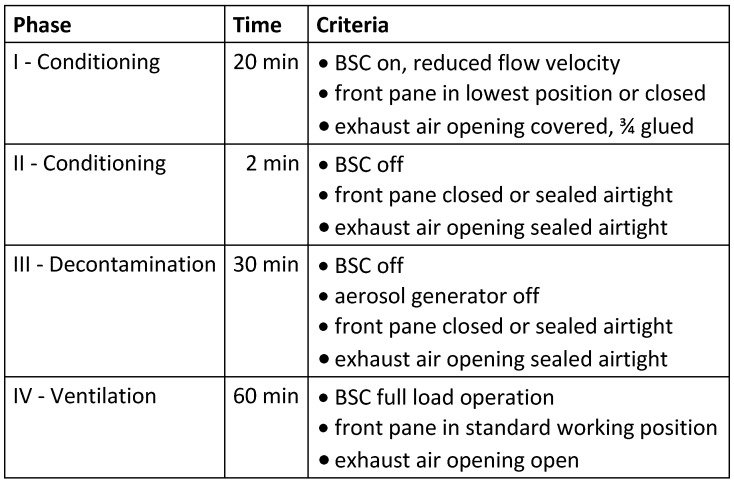
Phases and parameters of the process

**Table 2:**
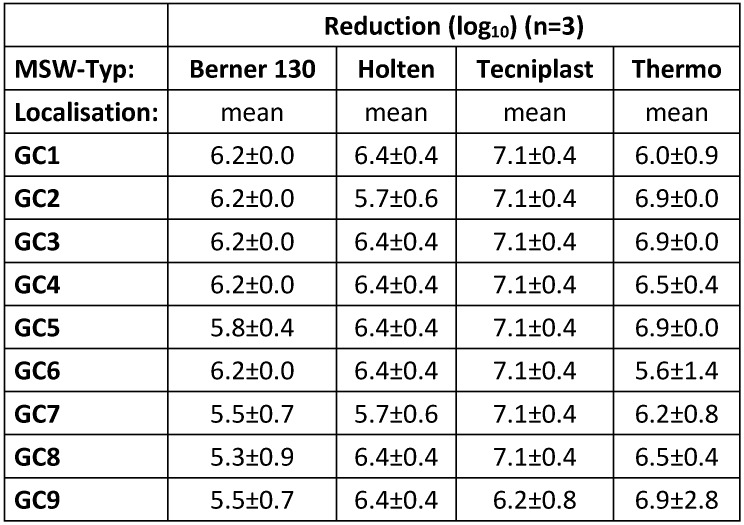
Inactivation data for different BSC types and biological indicator localisations; calculation of the reduction of infectivity according to [19]
